# Immunological Characterization of Chronic Nonbacterial Osteomyelitis (CNO) in Adults: A Cross‐Sectional Exploratory Study

**DOI:** 10.1002/jbm4.10818

**Published:** 2023-09-11

**Authors:** Anne T. Leerling, Elisabeth H. Andeweg, Juliette Faber, Trea C.M. Streefland, Olaf M. Dekkers, Natasha M. Appelman‐Dijkstra, Elizabeth M. Winter

**Affiliations:** ^1^ Department of Internal Medicine, Division of Endocrinology Leiden University Medical Center Leiden The Netherlands; ^2^ Center for Bone Quality Leiden University Medical Center Leiden The Netherlands; ^3^ Department of Clinical Epidemiology Leiden University Medical Center Leiden The Netherlands

**Keywords:** BIOCHEMICAL INFLAMMATION, BONE TURNOVER MARKERS, CHRONIC NON‐BACTERIAL OSTEOMYELITIS, CYTOKINES, SAPHO, SYSTEMIC INFLAMMATORY BASED SCORES

## Abstract

Chronic nonbacterial osteomyelitis (CNO) is a rare disease spectrum affecting children and adults. Adult CNO may occur as isolated bone inflammation, or with a broad range of extraskeletal features. CNO pathophysiology, including the key drivers of inflammation, remains largely unknown. For pediatric CNO, a role for pro‐inflammatory cytokine dysregulation has been proposed, but studies in adults are scarce. We therefore provide immunological characterization of adult CNO. Cross‐sectional study in our referral center including adult CNO patients (*n* = 172) and healthy controls (*n* = 65). Inflammation parameters and systemic inflammatory based scores(SIBS, including neutrophil/lymphocyte ratio [NLR] and systemic immune inflammation index [SII]) were compared between groups. Cytokine expression was explored with electrochemiluminescent immunoassays in 33 patients, eight healthy controls and 21 osteoporosis patients. Routine inflammation markers were higher in patients than in controls, but generally remained within reference range. Systemic inflammation was more pronounced in patients with additional vertebral involvement as compared to those osteitis in the anterior chest wall alone, in patients with comorbid pustulosis palmoplantaris or psoriasis, and in patients with strongly rather than moderately increased lesional uptake on nuclear imaging. SII was elevated in CNO patients too, but NLR was not. Cytokine expression was generally nondifferential between patients and both control groups, and patients displayed low absolute concentrations of pro‐inflammatory cytokines. In this adult CNO cohort, systemic inflammation was generally subtle, but more pronounced in patients with vertebral lesions, associated skin disease, and strongly increased uptake on nuclear imaging. SII was increased in patients compared to healthy controls. Contrasting pediatric studies, we found no increased expression of the pro‐inflammatory cytokines that have been proposed to drive the inflammatory cascade, like interleukin‐6, ‐8, and ‐17 (IL‐6, IL‐8, and IL‐17), and tumor necrosis α (TNF‐α). Further studies are needed to evaluate the use of SII in diagnosis and monitoring of CNO, and elucidate the role of cytokine dysregulation in adult disease. © 2023 The Authors. *JBMR Plus* published by Wiley Periodicals LLC. on behalf of American Society for Bone and Mineral Research.

## Graphical abstract

1. Generic inflammation parameters like ESR, CRP, platelet and neutrophil count are relatively increased in our adult CNO cohort compared to healthy controls, but stay within absolute reference range.
2. Within patients, vertebral involvement and skin inflammation are associated with more pronounced systemic inflammation as compared to isolated bone involvement of the anterior chest wall.
3. Systemic immune inflammation index (SII; product of platelet count and neutrophil/lymphocyte Ratio [NLR]) is elevated in adult CNO patients, as also seen in related rheumatic diseases.
4. The hypothesis proposing cytokine dysregulation as a fundamental component of adult CNO pathophysiology was not substantiated within the present cohort, which exhibited low levels of pro‐inflammatory cytokines.

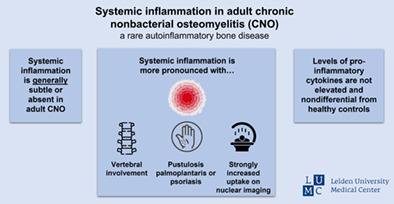

## Introduction

Chronic nonbacterial osteomyelitis (CNO) is a rare disease spectrum that occurs both in children and adults. The spectrum is characterized by relapsing–remitting bone inflammation causing osteosclerosis and hyperostosis.^(^ ^1^, ^2^ ^)^ Knowledge of CNO remains constrained: its pathophysiology is largely unknown and there are no evidence‐based therapeutics. Also, both pediatric and adult patients face diagnostic and therapeutic delay, which has been associated with high disease burden.^(^ ^3^, ^4^, ^5^ ^)^ Off‐label management for all CNO types varies from nonsteroidal anti‐inflammatory drugs (NSAIDs) to control pain and inflammation, and in case of failure, disease modifying anti‐rheumatic drugs (DMARDs), biologicals, corticosteroids and bisphosphonates.^(^ ^5^ ^)^

Adult CNO is indicated by a variety of names in current literature. Due to the typical localization of lesions in the anterior chest wall (ACW), the name sternocostoclavicular hyperostosis (SCCH) is used in several countries.^(^ ^5^ ^)^ CNO that occurs in combination with synovitis, pustulosis palmoplantaris (PPP), or acne is commonly indicated by the acronym synovitis, acne, pustulosis, hyperostosis, osteitis (SAPHO) syndrome, in which the letters H and O encompass the bone inflammation characteristic of CNO.

CNO is postulated to be have an auto‐inflammatory base, in which systemic immune activation and pro‐inflammatory cytokine imbalance lead to localized bone lesions. However, the supportive evidence for this auto‐inflammatory pathogenesis mainly derives from studies conducted in pediatric populations, and it remains unclear to what degree pediatric CNO resembles the adult variant. Clinically, children with CNO, also known as chronic recurrent multifocal osteomyelitis (CRMO), may present with systemic symptoms of inflammation like fever, subfebrile temperature, or weight loss, whereas in adults this is rarely seen.^(^ ^5^, ^6^ ^)^ Biochemically, mild to moderate elevation of generic inflammation markers like C‐reactive protein (CRP) and erythrocyte sedimentation rate (ESR) are common in children, but hardly seen in adults.^(^ ^7^, ^8^ ^)^ Likewise, pediatric cytokine analyses have revealed increased expression of pro‐inflammatory cytokines, specifically interleukin (IL)‐6, IL‐17A, and tumor necrosis factor α (TNFα). For adults, existing studies suggest increased expression of these cytokines too, additionally including IL‐8, but data remain scarce.^(^ ^9^, ^10^, ^11^, ^12^, ^13^, ^14^ ^)^

In sum, there is a need to further explore the auto‐inflammatory phenotype of adult CNO and assess whether it is reflected to the same degree as in children. Understanding auto‐inflammatory mechanisms and key drivers of disease is of major importance to make way for rational treatments in the future. We therefore provide an exploratory immunological characterization of adult CNO in a large cohort situated at our expert referral center. Apart from generic inflammation markers and cytokine profiles, we also include the novel parameters of systemic inflammatory‐based scores (SIBSs). These have emerged as sensitive reflectors of inflammation in other rheumatic diseases and are deployed as diagnostic predictors and disease monitoring biomarkers.^(^ ^15^, ^16^, ^17^ ^)^

## Patients and Methods

This study was approved by the Medical Ethical Review Board associated with the Leiden University Medical Center (LUMC) in the Netherlands. Informed consent was obtained from all participants for the clinical data collection, and subjects participating in the Biobank Bone Remodeling and Mineralization Disorders signed specific consent regarding the storage and additional analyses of their samples.

### Design and population

An exploratory single center, cross‐sectional retrospective study was conducted among adult patients referred to the expert referral center for CNO at the Leiden University Medical Center between 1992 and 2022 for suspected CNO (part of the recruited cohort has been reported elsewhere^(^ ^3^ ^)^). Conduct of the present study adhered to the Strengthening the Reporting of Observational Studies in Epidemiology (STROBE) guidelines for observational studies.^(^ ^18^ ^)^ In absence of validated classification criteria, diagnosis of CNO was based on multidisciplinary assessment, based upon the combination of chronic inflammatory bone pain and imaging features of osteitis, sclerosis, hyperostosis, and locally increased uptake on nuclear imaging (thus independent of biochemical parameters). For the aim of the study, referred patients were classified as CNO (in case diagnosis was confirmed) or healthy controls (in case no inflammatory pathology was found). CNO patients were further categorized into clinical subtypes according to skeletal distribution (involvement of the anterior chest wall [ACW], vertebrae, and mandible, or combinations) and extraskeletal features (pustulosis palmoplantaris [PPP] and/or psoriasis, arthritis, or both). Exclusion criteria were (i) absence of laboratory investigations performed at presentation (controls) or either at presentation or during active disease, as defined by inflammatory bone pain and increased uptake of bone lesions on nuclear imaging (patients); (ii) the use of immunomodulatory or antiresorptive medications ≤3 and ≤9 months respectively prior to laboratory investigation (apart from NSAIDs); (iii) infection at time of laboratory investigation; (iv) indefinite diagnosis of CNO; and (5) confirmed auto‐inflammatory pathology in controls. Due to the exploratory nature of the study, no prespecified endpoint was handled and sample size was not precalculated, according to the STROBE guidelines.

### Data collection

Biochemical data were extracted from the electronic health records, ie, ESR, CRP, alkaline phosphatase (ALP), serum cross‐linked C‐telopeptide of type I collagen (CTx), N‐terminal propeptide of type I procollagen (P1NP), and full blood count including leukocyte differential count. In case laboratory investigations were available at multiple time points for patients (eg, at presentation and during clinical flare‐up), data were collected from the most complete investigation. The following SIBS were calculated by dividing their counts (1 × 10^9^/L) as appropriate^(^ ^16^ ^)^: neutrophil/lymphocyte ratio (NLR), platelet to lymphocyte ratio (PLR), and lymphocyte to monocyte ratio (LMR). Also, the systemic immune‐inflammation index (SII) was determined by the multiplication of the platelet count and NLR.^(^ ^19^ ^)^ The following ancillary data were collected: demographic data, length and weight, medical and treatment history, intoxications, radiological reports. Visual analogue scales (VASs) for maximal and average pain measured with Brief Pain Inventory (BPI) and Shoulder Function Assessment Scale (SFA) were used as clinical measures of disease activity,^(^ ^20^, ^21^, ^22^ ^)^ whereas radiologic disease activity was defined by the degree of increased isotope uptake on technetium radiolabeled hydroxymethylene diphosphonate single positron emission computed tomography ([99mTc]Tc‐HDP‐SPECT/CT). Degree of uptake was classified as moderate or strong, as narratively indicated in radiology reports by nuclear medicine physicians with specific expertise in CNO.

### Cytokine analyses

Biobank Bone Remodeling and Mineralization Disorders collection started in 2015 in newly referred patients and those under continued clinical care. Biobank sampling is part of routine clinical care, independent of diagnosis. In the subset of included patients and healthy controls for whom biobank materials were available, cytokine profiles were analyzed (33 patients, 8 controls). Due to the limited number of control samples, a second control group was established consisting of osteoporosis patients, excluding those with auto‐inflammatory comorbidities, those suffering active infection at the time point of sampling, those taking immunomodulatory medications (apart from NSAIDs) and those who had sustained a fracture <6 weeks before sampling. Serum samples in the biobank are collected in nonfasting state before 15:00 CET and processed <4 hours after collection. A total of 500 μL serum is stored at −20°C immediately after processing and transferred to −80°C freezers within maximally 8 hours after collection.^(^ ^23^ ^)^ Serum levels of cytokines were analyzed using electrochemiluminescent immunoassays from Meso Scale Discovery, Rockville, MD, USA; IL‐1β, IL‐6, IL‐8, TNF‐α, IL‐10, IFN‐γ, IL‐12p70, IL‐13, IL‐2, and IL‐4 with V‐Plex Proinflammatory panel 1 (K15049D‐1) and IL‐17A, IL‐21, IL‐22, IL‐23, IL‐27, IL‐31, and macrophage inflammatory protein‐3 alpha (MIP‐3α) with using V‐Plex Plus Th17 Panel 1 Human Kit (K15085G‐1). All analyses were conducted according to the manufacturer's recommendations and without additional freeze/thaw cycles and with the eight‐point calibration curve, providing a dynamic range, analyzed in duplicate. In addition, serum levels of receptor activator of nuclear factor kappa beta (RANKL) and osteoprotegerin (OPG) were analyzed in duplicate by sandwich enzyme‐linked immunosorbent assay (ELISA) (R&D Systems, Minneapolis, MN, USA) using TRANCE/RANKL/TNFSF11 DUO Set (DY626) with 0.5‐fold dilution and using Recombinant Human Osteoprotegerin/TNFRSF11B (DY805) in undiluted samples, respectively.

### Statistical analysis

SPSS statistics version 25 (IBM Corp., Armonk, NY, USA) was used for statistical analyses. Results are presented as percentages for categorical data and mean ± standard deviation (SD) or median (interquartile range [IQR]) for continuous variables. Characteristics were evaluated for patients and controls using chi‐square tests for categorical data, unpaired *t* tests or one‐way analysis of variance (ANOVA) for parametric numerical and Mann‐Whitney *U* tests or Kruskal‐Wallis tests for nonparametric data. Subgroup analyses were performed in patients according to clinical subtype, degree of isotope uptake on nuclear imaging, NSAID use, age, and disease duration. Correlations between inflammation markers, SIBS, bone markers, and clinical measures of disease activity were evaluated in patients with Pearson's correlation coefficient or Spearman's rank correlation as appropriate. For the immunoassays, only analytes with at least 50% of measurements of each group within the detection range were statistically analyzed. If this analysis threshold was reached, the lower limit of detection (LLOD) multiplied by 0.5 was used for calculations on samples with measurements below the detection range.^(^ ^24^ ^)^ Grubb's test was used for detecting outliers in parametric data, whereas data points greater than 1.5 times the IQR above the 75th percentile were regarded outliers in nonparametric data^(^ ^25^ ^)^; outliers were excluded from analysis. Level of significance was set at *p* < 0.05, but Bonferroni correction was used to adjust for multiple testing in the correlation analyses.

## Results

**Fig. 1:**
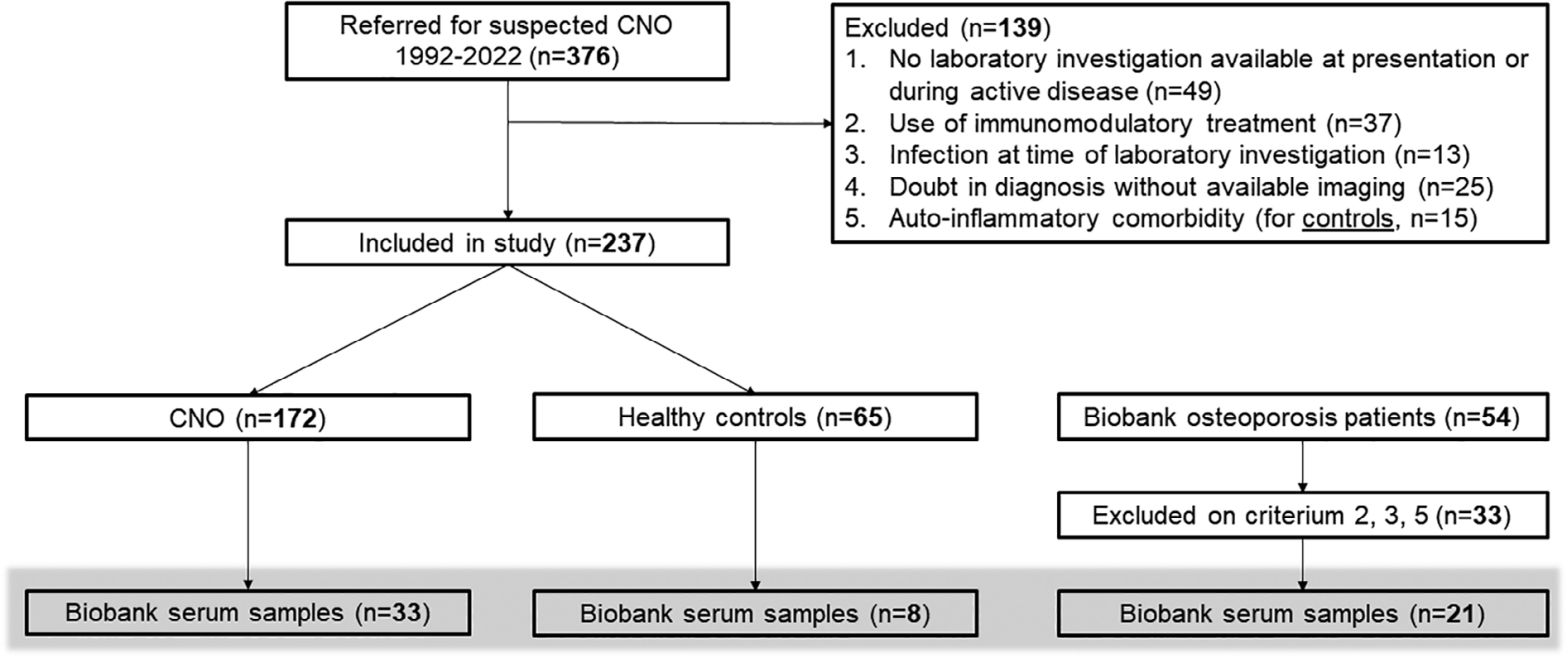
Overview of patient inclusion.

A total of 376 patients were referred to the outpatient clinic for suspected CNO between 1992 and 2022. After application of exclusion criteria, 182 CNO patients and 65 healthy controls could be included in the study, of whom 33 and 8 had serum samples available for cytokine profiling respectively (see Fig. 1).

Both CNO patients and controls were predominately female (91.2% and 84.6%, *p* = 0.136, Table 1), but patients were younger at time of presentation compared to controls (44.8 ± 13.0 versus 51.1 ± 12.7 years, *p* = 0.001). Daily NSAID use was slightly higher amongst patients (39.2% versus 26.6%, *p* = 0.074). Mean diagnostic delay and disease duration for patients were 5.8 years (range, 0–30 years) and 7.8 years (range, 0–41 years). Categorizing patients according to skeletal distribution, 80.8% had CNO of the ACW only, and smaller patient groups presented with vertebral and mandibular involvement. Considering extraskeletal features, 30.2% suffered from PPP and/or psoriasis, 4.1% from arthritis, and 5.2% from both. Still, the majority of patients (60.5%) did not display extraskeletal features. Of the patients with ACW involvement only, 35% exhibited extraskeletal features of arthritis and/or PPP/psoriasis, compared to 58% in patients with ACW and vertebral/mandibular involvement (*p* = 0.028). Controls were diagnosed with osteoarthritis (51%), functional complaints (34%), rotator cuff injury (3%), sternoclavicular dislocation (3%), or diagnosis remained unknown (9%).

**Table 1:** Clinical Characteristics of CNO Patients and Healthy Controls

	CNO (*n* = 172)	Controls (*n* = 65)	*p*
Gender, female, *n* (%)	157 (91.2)	55 (84.6)	0.136
Age (years), mean ± SD	44.8 ± 13.0	51.1 ± 12.7	**0.001**
Active smoker, *n* (%)	51 (29.8)	15 (23.8)	0.894
Body mass index (kg/m^2^), mean ± SD	26.2 ± 5.2	27.0 ± 5.7	0.417
Daily NSAID use, *n* (%)	67 (39.2)	17 (26.6)	0.074
Diagnostic delay (years)	5.8 (range, 0–30)		
Disease duration (years)	7.8 (range, 0–41)		
Auto‐inflammatory comorbidity (any), *n* (%)	76 (44.2)		
Clinical subtype n (%) Skeletal distribution pattern			
ACW only	139 (80.8)		
ACW + vertebrae	15 (8.7)		
Vertebrae only	2 (0.8)		
ACW + mandible	14 (8.1)		
ACW + vertebrae + mandible	2 (0.8)		
Extraskeletal features			
PPP and/or psoriasis	52 (30.2)		
Arthritis	7 (4.1)		
PPP and/or psoriasis + arthritis	9 (5.2)		
None	104 (60.5)		

Abbreviations: ACW = anterior chest wall; CNO = chronic nonbacterial osteomyelitis; NSAID = nonsteroidal anti‐inflammatory drug; SD = standard deviation; PPP = palmoplantar pustulosis.

Generic inflammation markers of ESR, CRP, platelet count, WBC, and neutrophils were significantly higher in patients compared to healthy controls, though their absolute values were within or slightly above reference range (see Table 2). Patients more frequently showed values of ESR and CRP above reference range (32% versus 16%, and 40% versus 5%, respectively). Concerning SIBS, SII, a score based on platelet count and NLR, was also higher amongst CNO patients compared to controls (mean difference 160.5, 95% confidence interval [CI] 18.1–302.9, *p* = 0.028). No differences between groups were found for NLR, PLR, and LMR. Regarding bone markers, AP and P1NP were nondifferential between groups and elevated above reference range in similar proportions of subjects. Serum CTx was slightly lower in CNO patients but still mostly within normal range.

**Table 2:** Generic Inflammation Markers, SIBS, and Bone Markers in CNO and Healthy Controls

Generic inflammation markers	CNO (*n* = 172)	Healthy controls (*n* = 65)	*p*
ESR (mm/h, ref. <20)	12.0 (6.0–28.0)	9.0 (2.0–14.0)	0.001
> ref, *n* (%)	50 (32)	9 (16)	0.018
CRP (mg/L, ref <5)	3.7 (1.0–6.9)	1.0 (0.9–2.4)	<0.001
> ref, *n* (%)	44 (40)	2 (5)	<0.001
Platelets (×10^3^/μL, ref 150–400)	285.0 (244.0–330.0)	249.0 (205.0–297.2)	0.002*
WBC (×10^3^/μL, ref 4–10)	7.6 (6.1–9.6)	6.8 (5.8–7.7)	0.004*
Neutrophils (×10^3^/μL, ref 1.5–7.5)	4.6 (3.6–6.6)	4.1 (3.2–5.0)	0.031*
Lymphocytes (×10^3^/μL, ref 1–3.5)	2.2 (1.9–2.5)	2.2 (1.8–2.8)	1.000*
Monocytes (×10^3^/μL, ref 0.1–1)	0.6 (0.5–0.7)	0.6 (0.5–0.7)	0.667

SIBS	CNO (*n* = 72)	Healthy controls (*n* = 25)	*p*
NLR	2.1 (1.8–2.6)	1.8 (1.5–2.7)	0.140
PLR	134.7 (106.2–172.4)	122.0 (94.8–154.4)	0.171
LMR	3.9 (2.9–5.1)	4.3 (2.9–5.1)	0.754
SII	658.8 (482.5–840.1)	527.4 (390.0–691.0)	0.028*

Bone markers	CNO (*n* = 138)	Healthy controls (*n* = 56)	*p*
ALP (U/L, ref <98)	80.0 (62.5–95.5)	74.5 (60.5–92.8)	0.772*
> ref, *n* (%)	32 (20)	11 (20)	0.970
P1NP (μg/L, ref <59)	44.0 (31.0–58.3)	47.0 (33.5–61.5)	0.328*
> ref, *n* (%)	34 (25)	18 (32)	0.319
CTx (μg/L, ref <0.573)	0.214 (0.158–0.326)	0.263 (0.187–0.389)	0.054
> ref, *n* (%)	3 (2)	5 (9)	0.033

*Note*: Data presented as median (IQR).

Abbreviations: ALP = alkaline phosphatase; CI = confidence interval; CRP = C‐reactive protein; CTx = beta‐crosslaps; ESR = erythrocyte sedimentation rate; LMR = lymphocyte‐monocyte ratio; ref = reference range; NLR = neutrophil‐lymphocyte ratio; P1NP = procollagen 1 N‐terminal propeptide; PLR = platelet‐lymphocyte ratio; SII = Systemic immune Inflammation Index; WBC = white blood cell.

* Parametric test used for comparative analysis.

**Fig. 2:**
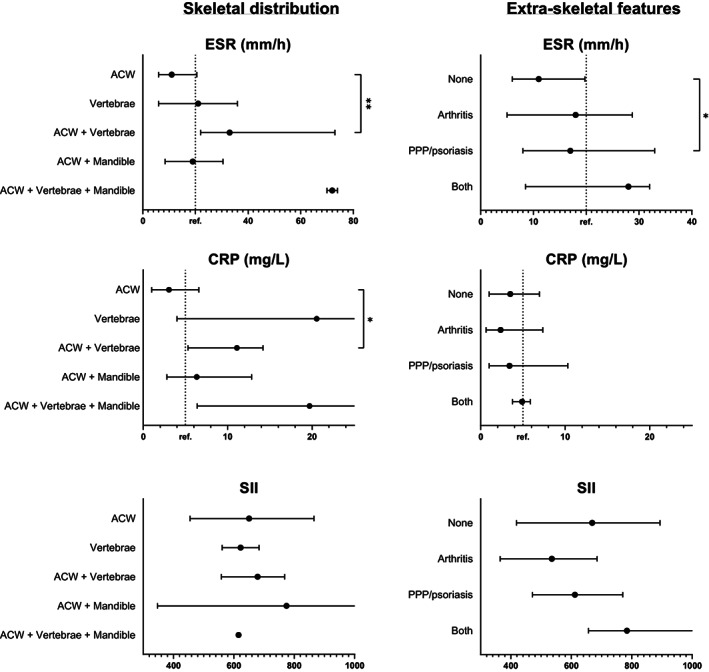
ESR, CRP, and SII stratified for skeletal distribution (left) and extraskeletal involvement (right) (data presented as median, interquartile range). **p < 0.01, *p < 0.05.

Subgroup analyses for biochemical parameters are displayed in Fig. 2 and Table S1. ESR and CRP were higher in patients with ACW + vertebral involvement, as compared to ACW involvement alone (median [IQR] 33 [22–73] versus 11 [6–21] mm/hr for ESR, *p* < 0.01 and 11^(^ ^5^, ^6^, ^7^, ^8^, ^9^, ^10^, ^11^, ^12^, ^13^, ^14^ ^)^ versus 3.1^(^ ^1^, ^2^, ^3^, ^4^, ^5^, ^6^, ^7^ ^)^ mg/L for CRP, *p* = 0.024, Fig. 2). No significant differences in ESR and CRP were found between ACW + mandibular involvement and ACW involvement alone. SII was similar for the different skeletal distribution types. As for extraskeletal features, presence of PPP and/or psoriasis was associated with higher ESR (median [IQR] 17^(^ ^8^, ^9^, ^10^, ^11^, ^12^, ^13^, ^14^, ^15^, ^16^, ^17^, ^18^, ^19^, ^20^, ^21^, ^22^, ^23^, ^24^, ^25^, ^26^, ^27^, ^28^, ^29^, ^30^, ^31^, ^32^, ^33^ ^)^ versus 11^(^ ^6^, ^7^, ^8^, ^9^, ^10^, ^11^, ^12^, ^13^, ^14^, ^15^, ^16^, ^17^, ^18^, ^19^, ^20^ ^)^ mm/hr, *p* = 0.026), but this was not the case for CRP or SII. Stratified according to the degree of isotope uptake on [99mTc]Tc‐HDP‐SPECT/CT, patients with strongly increased uptake had higher CRP levels compared to those with moderately increased uptake, and also exceeded reference range more frequently (29% versus 9%, *p* = 0.007, Table S1), but no differences in bone markers were detected. NSAID use, age at laboratory investigation, or disease duration did not affect inflammation parameters, SIBS, or bone markers (Table S1).

Correlations between inflammation and bone markers and clinical measures of disease activity are depicted in Table 3. We found no correlations between inflammation markers ESR, CRP, NLR, and SII and VAS scores for maximal or average pain or shoulder function. Associations between inflammation markers and bone markers were mostly absent too, except for a correlation between ESR and CRP with ALP (0.326 and 0.349, *p* < 0.001 [below significance level adjusted by Bonferroni correction 0.0016]).

**Table 3:** Correlations Between Inflammation and Bone Markers and Disease Activity in CNO Patients: Pearson's Correlation Coefficient or Spearman's Rank Depending on Normality

	ESR	CRP	NLR	SII	P1NP	CTx	ALP	VAS max	VAS average	SFA total
ESR	–	NA	0.102	0.358*	0.090	0.165	0.326**	−0.105	−0.016	0.092
CRP	NA	–	0.027	0.454**	0.068	0.149	0.349**	−0.049	0.052	−0.048
NLR	–	–	–	NA	−0.122	−0.169	−0.004	−0.293	−0.416	−0.030
SII	–	–	–	–	−0.084	−0.116	0.242	−0.361	−0.287	−0.051

*Note*: *p < 0.01, **p < 0.0016 (significance level adjusted by Bonferroni correction).

Abbreviations: CRP = C‐reactive protein; CTx = beta‐crosslaps; ESR = erythrocyte sedimentation rate; NA = not applicable; NLR = neutrophil‐lymphocyte ratio; P1NP = procollagen 1 N‐terminal propeptide; SFA = shoulder function assessment; SII = Systemic immune Inflammation Index; VAS = visual analogue scale (pain).

### Cytokine and RANKL/OPG analyses

**Fig. 3:**
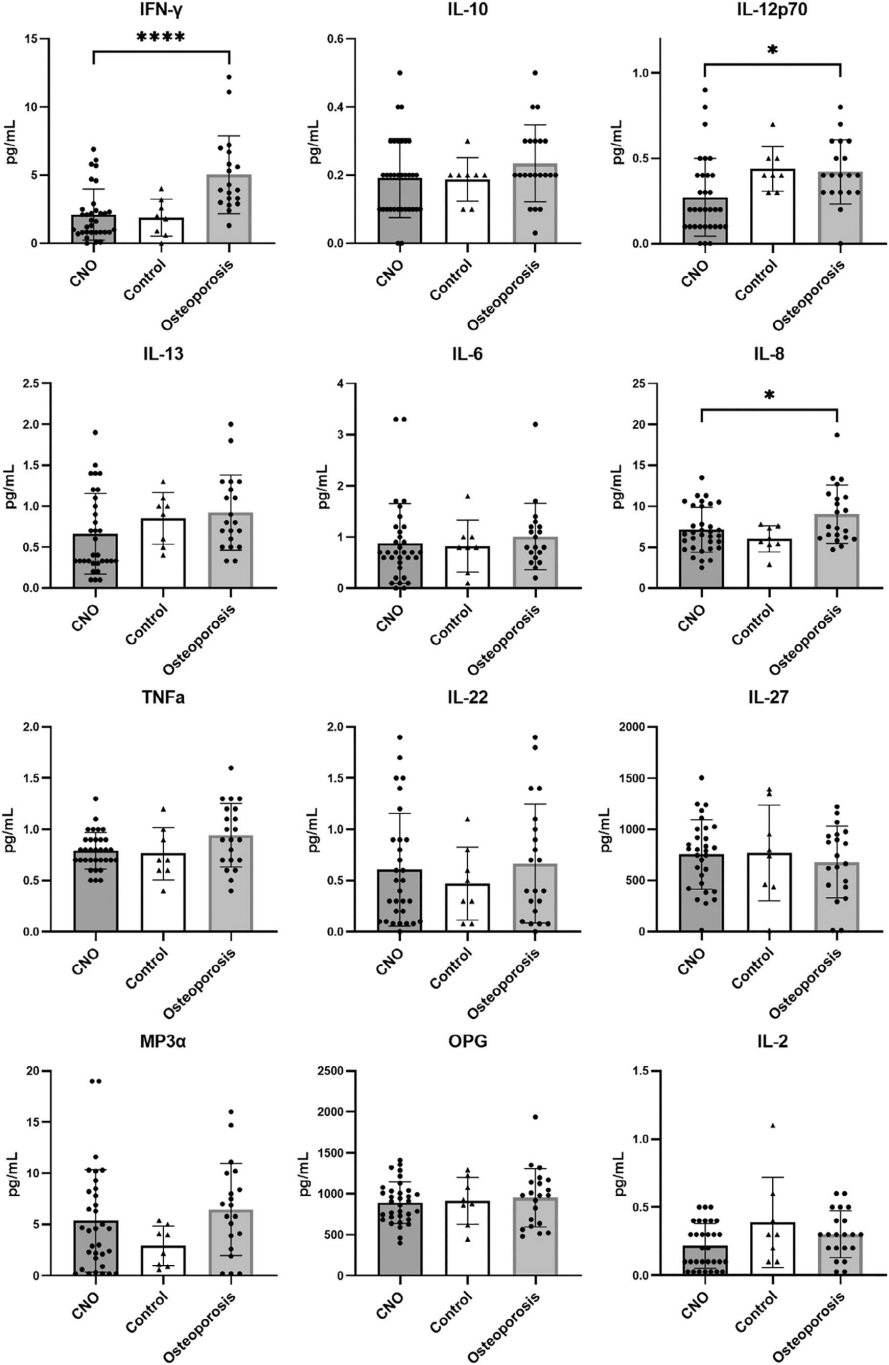
Serum levels pro‐ and anti‐inflammatory cytokines in CNO patients, healthy controls, and osteoporosis patients. *p < 0.05, ****p < 0.0001.

**Fig. 4:**
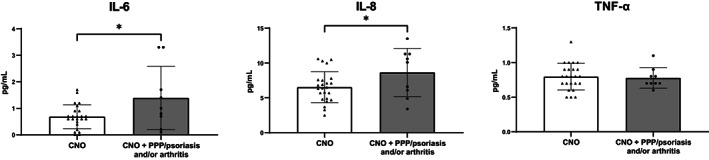
Serum levels of IL‐6, IL‐8, and TNF‐α stratified for CNO without extraskeletal involvement versus CNO with PPP/psoriasis and/or arthritis. *p < 0.05.

A total of 33 patients, eight healthy controls, and 21 osteoporosis patients were included for cytokine and RANKL/OPG analyses, of whom clinical characteristics are displayed in Table S2. IL‐1β, IL‐4, IL‐21, IL‐31, IL‐17A, IL‐23, and RANKL were excluded from quantitative analysis because <50% of the measurements per group were within detection range. For remaining cytokines, no significant differences were found between groups for IL‐2, IL‐10, IL‐13, IL‐6, TNF‐α, IL‐22, IL‐27, and MIP‐3α. Generally, cytokine levels in CNO patients were low in the absolute sense, including those of IL‐6, IL‐8, and TNF‐α (mean 0.9 ± 0.8, 7.1 ± 2.7, and 0.8 ± 0.2 pg/mL, respectively) (see Fig. 3, complete numerical results available in Table S3). IFN‐γ and IL‐12p70 were higher osteoporosis patients than in patients (5.0 ± 2.8 versus 2.1 ± 1.9 [*p* < 0.001] and 0.4 ± 0.1 versus 0.3 ± 0.2 pg/mL [*p* = 0.021]). Likewise, IL‐8 was found higher in osteoporosis patients as compared to patients (9.0 ± 4.0 versus 7.2 ± 2.7 pg/mL, *p* = 0.022). OPG levels were similar between patients, healthy controls, and osteoporosis patients. Given that elevated levels of IL‐6, IL‐8, and TNF‐α were previously found in CNO cohorts with higher prevalence of arthritis and skin conditions as compared to the present cohort, stratified analysis was performed for these cytokines according to the presence of these additional features (Fig. 4). Patients with CNO with associated PPP/psoriasis and/or arthritis demonstrated higher IL‐6 and IL‐8 levels than patients without extraskeletal features (mean difference 0.7 pg/mL [95% CI] 0.2–1.3, *p* = 0.02 and 2.1 pg/mL [95% CI] 0.03–4.2, *p* = 0.047, Fig. 4), but this was not the case for TNF‐α. Comparing patients with daily NSAID use and those not using NSAIDs, no statistically significant differences were observed for any of the analytes (data not shown).

## Discussion

CNO is a rare bone disease spectrum, of which pathophysiology is yet limitedly understood. Current literature proposes an auto‐inflammatory base for pediatric and adult CNO, supported by the high prevalence of comorbidities like arthritis and autoimmune skin disease,^(^ ^2^, ^26^ ^)^ and the fact that immunosuppressive therapy seems clinically effective. Biochemically, the supportive evidence for auto‐inflammation as the main driver of CNO mostly derives from pediatric populations, especially regarding cytokine dysregulation.^(^ ^2^, ^5^ ^)^ We therefore explored biochemical immunological parameters, including SIBS and cytokine profiles in adults with CNO compared to healthy controls and osteoporosis patients.

In line with previous studies, we found that generic inflammation parameters such as ESR, CRP, and platelet count are relatively increased in adult CNO, though generally remain within reference range.^(^ ^1^ ^)^ Our data, therefore, are likely to be generalizable. Also, this finding holds relevance as adult CNO currently lacks established diagnostic criteria and validated disease activity indices. In order to facilitate their future development, it is crucial not only to identify markers that accurately reflect diagnosis and disease activity but also to recognize those that are unsuitable for this purpose. Remarkably, the between‐patient variation of inflammation parameters was substantial, suggesting the full spectrum of CNO is clinically heterogeneous. Subdividing patients according to skeletal distribution pattern, patients with bone involvement outside the ACW, especially the vertebrae, displayed more pronounced biochemical inflammation than those with ACW lesions alone. ESR was also slightly higher in patients with PPP and/or psoriasis or arthritis, indicating that biochemical inflammation may surface only with sufficient systemic involvement. This finding is relevant as it highlights the diagnostic challenges in patients with limited disease. When CNO exhibits as osteitis of the ACW alone—as proves the most frequent clinical subtype in the present study—symptoms may already lack specificity due to the absence of distinctive skin and joint involvement, and diagnosis is further complicated by the relatively unremarkable laboratory results. This emphasizes the critical importance of advancing early disease biomarkers, possibly radiological markers, to facilitate prompt diagnosis, especially in less apparent cases.

This study also unprecedentedly evaluated the relatively novel SIBS in adult CNO. SIBS have emerged as promising biomarkers in assessing low‐grade systemic inflammation in other musculoskeletal auto‐immune diseases such as rheumatoid arthritis, psoriatic arthritis, and ankylosing spondylitis, of which the latter two may clinically overlap with adult CNO.^(^ ^27^ ^)^ Specifically, NLR, PLR, and SII have been found to differentiate between patients and controls, and correlate with disease activity.^(^ ^15^, ^16^, ^17^, ^19^, ^28^, ^29^ ^)^ We found no differences in NLR and PLR between CNO patients and healthy controls, but our patient group displayed NLR and PLR values comparable to those reported for rheumatoid arthritis, ankylosing spondylitis, and connective tissue diseases.^(^ ^16^, ^17^, ^29^ ^)^ SII, the product of platelet count and NLR, was significantly higher in CNO patients compared to controls. Although SIBS are not specific for diagnosis of CNO, and rather reflect a general state of immune activation, their clinical utility for diagnosis and monitoring deserves further study, especially considering other generic markers seem of limited use, and SIBS are easily obtainable and economical.

Regarding cytokine profiles, our data do not support the suspected pro‐inflammatory dysregulation that has been proposed in pediatric, and a handful of adult CNO studies.^(^ ^9^, ^10^, ^11^, ^12^, ^14^, ^30^ ^)^ In general, the cytokines assessed exhibited no significant differences between patients and controls, and notably, most cytokines also displayed low absolute values amongst patients. Several cytokines were not previously examined in the context of CNO, making this study a first measurement. Conversely, IL‐6, IL‐8, TNF‐α, and IL‐17A were evaluated in prior studies and our findings contrast their results. IL‐6 has been found elevated in multiple pediatric CNO cohorts,^(^ ^9^, ^10^, ^31^ ^)^ and also demonstrated higher concentrations in two studies on adults with SAPHO syndrome.^(^ ^12^, ^14^ ^)^ The same holds true for IL‐8, TNF‐α, and IL‐17A, of which the previously published means of 36.41 pg/mL, 19–25.2 pg/mL, and 29–83 pg/mL vastly exceed those found in the current study. Although we found significantly higher levels of IL‐6 and IL‐8 in CNO patients with additional skin and/or joint features, these too were still markedly lower than previously reported.^(^ ^9^, ^14^, ^30^ ^)^

Thus, the present study did not confirm the hypothesis of dysregulated pro‐inflammatory cytokines in adult CNO. Although our study resembles previous one in terms of sample handling and the inclusion of patients with significant clinical and radiologic disease activity, several differences may explain our contrasting findings. First, the proportion of patients suffering from lesions outside the anterior chest wall and skin and joint features was considerably smaller in our cohort than in previous studies, and these features likely elevate overall inflammatory activity. Second, it is yet unknown autoinflammation merely triggers, or also sustains CNO. Although all included patients had active disease, mean disease duration of 7.3 years may have been longer than in previous studies, in which duration was 2 years or not reported.^(^ ^12^, ^14^, ^32^ ^)^ This subset might therefore have transitioned into a chronic phase of disease in which inflammation is no longer as systemically detectable. Third, the hypothesis of cytokine dysregulation in CNO rests mostly on pediatric data, and our results prompt the speculation whether this mechanism is also applicable to adult cases. Fourth, it is known that some cytokines are not stable at −80°C for longer than 2–4 years, and average storage time in our study was 4 years.^(^ ^33^ ^)^ However, this seems not to have affected our results as analyses yielded no different results when excluding samples stored >3 years. We may also have failed to detect differences due to lack of power. Nevertheless, the size of existing studies was not markedly larger, and we could also not detect trends that supposedly could have reached statistical significance in larger samples. All in all, our data warrant further studies in well‐balanced, larger patient groups to improve the external validity of the outcomes.

Apart from immunological parameters, our analyses focused on markers of bone metabolism, as CNO is associated with locally upregulated bone metabolism.^(^ ^5^, ^34^, ^35^, ^36^ ^)^ Our results did not confirm this pathophysiological process at a biochemical level; we found no clinically relevant differences between patients and controls for ALP, P1NP, and CTx because all patients using bisphosphonates—a common CNO treatment at our center—≤9 months were excluded in this study, the lower CTx levels in patients were not a result of therapy but may be due to patients being younger. Although a previous study found higher mean CTx levels in patients with active disease and a significant correlation with pain levels, we did not observe a correlation of bone turnover markers with clinical (pain) or radiologic disease activity.^(^ ^37^ ^)^ For RANKL and OPG, only one study so far has evaluated these markers in CNO, and found higher RANKL levels in patients with active disease compared to controls.^(^ ^14^ ^)^ In the present study, RANKL was <LLOD (78.1 pg/mL) in the majority of patients and controls, and OPG levels were similar between all groups. Further study is thus needed to resolve whether CNO is too local a disease to cause systemic disruption of bone parameters, and what their clinical relevance may be.

In conclusion, this adult CNO cohort showed a relative, but not absolute increase in generic inflammation markers as well as SII. This marker has proven valuable as a diagnostic and monitoring tool in other rheumatic diseases. Its clinical use in CNO should therefore be evaluated in future research. The present study found no pro‐inflammatory cytokine profiles to support systemic cytokine imbalance in adult CNO. Further studies are needed to evaluate the hypothesized cytokine dysregulation in the CNO spectrum, and to resolve whether it applies as much to the adult as the pediatric subtype.

## Author Contributions

**Anne T. Leerling:** Conceptualization; data curation; formal analysis; methodology; writing – original draft; writing – review and editing. **Elisabeth H. Andeweg:** Data curation; formal analysis. **Juliette Faber:** Data curation. **Trea C.M. Streefland:** Data curation; methodology. **Olaf M. Dekkers:** Methodology; writing – review and editing. **Natasha M. Appelman‐Dijkstra:** Writing – review and editing. **Elizabeth M. Winter:** Conceptualization; project administration; supervision; writing – review and editing.

## Disclosures

The authors declare no competing interests pertaining to the current study.

### Peer Review

The peer review history for this article is available at https://www.webofscience.com/api/gateway/wos/peer‐review/10.1002/jbm4.10818.

## Supporting information


**Table S1.** Subgroup analysis of biochemical parameters in CNO patients.

**Table S2.** Clinical characteristics of cohort subset evaluated for cytokine profiles and RANKL/OPG.

**Table S3.** Results of immunoassays for Th1/2 and Th17‐derived cytokines and ELISA for RANKL and OPG in CNO patients, healthy controls and osteoporosis patients.

## Data Availability

The datasets generated during and/or analyzed during the current study are available from the corresponding author on reasonable request.
