# Supplementary material for: Immunological Characterization of Chronic Nonbacterial Osteomyelitis (CNO) in Adults: A Cross‐Sectional Exploratory Study
Source: JBMR Plus. 2023 Sep 11;7(12):e10818. doi: 10.1002/jbm4.10818 (PMC10731106; doi:10.1002/jbm4.10818)
Supplement: Supplementary file 1 — Table S1. Subgroup analysis of biochemical parameters in CNO patients. [file JBM4-7-e10818-s001.docx]

**Supplementary table 1:** Subgroup analysis of biochemical parameters in CNO patients

|  | **Moderately increased uptake (n=32)** | **Strongly increased uptake (n=140)** | ***p*** |
| --- | --- | --- | --- |
| ESR (mm/h, ref. <20) | 11.0 (2.0 - 22.0) | 14.0 (6.0 - 28.0) | 0.079 |
| > reference range n (%) | 8 (25.0) | 42 (30.0) | 0.390 |
| CRP (mg/L, ref <5) | 3.0 (1.0 - 4.0) | 4.0 (1.0 - 8.0) | 0.044 |
| > reference range n (%) | 3 (9.4) | 41 (29.2) | 0.007 |
| Platelets (×10^3^/μL, ref 150-400) | 265.5 (229.8 - 304.0) | 291.0 (249.0 - 337.0) | 0.049* |
| WBC (×10^3^/μL, ref 4-10) | 7.5 (5.9 - 8.9) | 7.6 (6.1 - 9.7) | 0.754* |
| Neutrophils (×10^3^/μL, ref 1.5-7.5) | 4.0 (3.4 - 5.7) | 4.8 (3.9 - 7.2) | 0.092* |
| Lymphocytes (×10^3^/μL, ref 1-3.5) | 2.1 (1.8 - 2.4) | 2.2 (1.9 - 2.8) | 0.206* |
| Monocytes (×10^3^/μL, ref 0.1-1) | 0.5 (0.4 - 0.7) | 0.6 (0.5 - 0.7) | 0.205 |
| NLR | 2.1 (1.8 - 2.3) | 2.2 (1.8 - 2.8) | 0.432 |
| PLR | 131.9 (106.5 - 160.1) | 136.5 (105.3 - 181.3) | 0.814 |
| LMR | 4.0 (2.9 - 5.2) | 3.9 (3.0 - 5.2) | 0.892 |
| SII | 577.8 (380.3 - 706.8) | 705.6 (561.1 - 935.8) | 0.030* |
| ALP (U/L, ref <98) | 78.0 (63.3 - 97.0) | 81.0 (62.0 - 95.0) | 0.860* |
| P1NP (µg/L, ref <59) | 37.0 (31.0 - 55.0) | 45.0 (31.3 - 59.0) | 0.595* |
| CTx (µg/L, ref <0.573) | 0.193 (0.150 - 0.283) | 0.220 (0.159 - 0.333) | 0.510 |

***A:*** *Stratified for moderate versus strong increase in isotope uptake on nuclear imaging, reflecting disease activity*

Legend: CI, confidence interval; CTx, beta-crosslaps; ESR, erythrocyte sedimentation rate; CRP, C-reactive protein; NLR, neutrophil-lymphocyte ratio; PLR, platelet-lymphocyte ratio; P1NP, procollagen 1 N-terminal propeptide; SII, Systemic immune Inflammation Index.
data reported as median (IQR), * parametric test used for comparative analysis

|  | **Daily NSAID use (n=67)** | **No daily NSAID use (n=105)** | ***p*** |
| --- | --- | --- | --- |
| ESR (mm/h, ref. <20) | 11.0 (6.0 - 20.0) | 11.0 (6.0 - 22.8) | 0.340 |
| > reference range n (%) | 16 (23.9) | 31 (29.5) | 0.407 |
| CRP (mg/L, ref <5) | 3.4 (1.0 - 6.6) | 2.2 (1.0 - 5.5) | 0.460 |
| > reference range n (%) | 18 (26.9) | 22 (21.0) | 0.609 |
| Platelets (×10^3^/μL, ref 150-400) | 282.0 (233.5 - 330.5) | 268.0 (228.0 - 317.5) | 0.153* |
| WBC (×10^3^/μL, ref 4-10) | 7.64 (6.325 - 10.06) | 7.08 (5.87 - 8.31) | 0.216* |
| Neutrophils (×10^3^/μL, ref 1.5-7.5) | 4.86 (3.365 - 6.85) | 4.38 (3.5 - 5.22) | 0.582* |
| Lymphocytes (×10^3^/μL, ref 1-3.5) | 2.285 (1.86 - 2.775) | 2.19 (1.86 - 2.48) | 0.430* |
| Monocytes (×10^3^/μL, ref 0.1-1) | 0.59 (0.47 - 0.67) | 0.57 (0.43 - 0.7) | 0.703 |
| NLR | 2.14 (1.657 - 2.837) | 2.04 (1.66 - 2.58) | 0.885 |
| PLR | 131.5 (104.365 - 156.378) | 127.2 (106.0 - 167.1) | 0.514 |
| LMR | 4.2836 (3.2004 - 4.8305) | 3.86 (2.78 - 5.22) | 0.786 |
| SII | 661.4 (471.9 - 864.8) | 574.5 (405.2 - 748.8) | 0.339* |
| ALP (U/L, ref <98) | 73.5 (59.0 - 92.25) | 79.0 (62.3 - 97.0) | 0.736* |
| P1NP (µg/L, ref <59) | 40.0 (28.25 - 53.0) | 48.5 (35.0 - 60.8) | 0.040* |
| CTx (µg/L, ref <0.573) | 0.219 (0.140 – 0.327) | 0.231 (0.172 – 0.343) | 0.597 |

***B:*** *Stratified for NSAID use*

Legend: CI, confidence interval; CTx, beta-crosslaps; ESR, erythrocyte sedimentation rate; CRP, C-reactive protein; NLR, neutrophil-lymphocyte ratio; PLR, platelet-lymphocyte ratio; P1NP, procollagen 1 N-terminal propeptide; SII, Systemic immune Inflammation Index.
data reported as median (IQR), * parametric test used for comparative analysis

|  | **Age ≤40 (n=70)** | **Age 41-50 (n=37)** | **Age ≥51 (n=65)** | ***p*** |
| --- | --- | --- | --- | --- |
| ESR (mm/h, ref. <20) | 11.0 (6.0 - 22.0) | 11.0 (5.0 - 22.0) | 17.0 (6.0 - 33.0) | 0.093 |
| > reference range n (%) | 17 (26.7) | 11 (31.2) | 22 (40.0) | 0.293 |
| CRP (mg/L, ref <5) | 3.0 (1.0 - 6.8) | 5.4 (3.2 - 7.3) | 3.7 (1.0 - 8.4) | 0.330 |
| > reference range n (%) | 16 (33.3) | 12 (57.1) | 16 (35.6) | 0.151 |
| Platelets (×10^3^/μL, ref 150-400) | 285.0 (240.0 - 337.5) | 295.0 (255.0 - 341.0) | 281.0 (243.5 - 327.0) | 0.647* |
| WBC (×10^3^/μL, ref 4-10) | 7.7 (6.2 - 9.2) | 7.5 (6.1 - 10.3) | 7.3 (5.9 - 9.3) | 0.716* |
| Neutrophils (×10^3^/μL, ref 1.5-7.5) | 4.5 (3.3 - 6.9) | 5.5 (3.8 - 7.4) | 4.7 (3.5 - 6.4) | 0.641* |
| Lymphocytes (×10^3^/μL, ref 1-3.5) | 2.2 (1.9 - 2.5) | 2.2 (1.8 - 3.0) | 2.2 (1.8 - 2.7) | 0.826* |
| Monocytes (×10^3^/μL, ref 0.1-1) | 0.5 (0.4 - 0.6) | 0.5 (0.4 - 0.8) | 0.6 (0.5 - 0.7) | 0.214 |
| NLR | 1.9 (1.6 - 2.6) | 2.3 (1.8 - 3.0) | 2.1 (1.8 - 2.6) | 0.656 |
| PLR | 123.4 (107.7 - 157.4) | 136.5 (92.3 - 160.7) | 142.8 (108.1 - 183.0) | 0.592 |
| LMR | 4.0 (3.2 - 5.5) | 4.3 (2.9 - 5.0) | 3.8 (2.9 - 5.1) | 0.445 |
| SII | 609.2 (413.9 - 809.5) | 706.8 (490.7 - 849.7) | 669.2 (512.6 - 864.8) | 0.700* |
| ALP (U/L, ref <98) | 73.5 (56.8 - 89.2) | 81.0 (64.8 - 98.3) | 86.5 (72.0 - 99.8) | 0.011* |
| P1NP (µg/L, ref <59) | 45.0 (31.2 - 53.0) | 41.0 (27.0 - 61.3) | 44.0 (31.5 - 67.0) | 0.729* |
| CTx (µg/L, ref <0.573) | 0.200 (0.161 - 0.308) | 0.205 (0.139 - 0.395) | 0.226 (0.160 - 0.354) | 0.808 |

***C:*** *Stratified for age at laboratory investigation*Legend: CI, confidence interval; CTx, beta-crosslaps; ESR, erythrocyte sedimentation rate; CRP, C-reactive protein; NLR, neutrophil-lymphocyte ratio; PLR, platelet-lymphocyte ratio; P1NP, procollagen 1 N-terminal propeptide; SII, Systemic immune Inflammation Index.
data reported as median (IQR), * parametric test used for comparative analysis

|  | **0-4 years (n=73)** | **5-10 years (n=46)** | **≥ 11 years (n=36)** | ***p*** |
| --- | --- | --- | --- | --- |
| ESR (mm/h, ref. <20) | 11.0 (6.0 - 19.0) | 17.0 (9.0 - 28.0) | 14.0 (6.0 - 31.0) | 0.191 |
| > reference range n (%) | 14 (21.5) | 18 (41.9) | 13 (41.9) | 0.038 |
| CRP (mg/L, ref <5) | 3.0 (1.0 - 6.9) | 4.4 (3.0 - 6.6) | 4.8 (1.0 - 11.1) | 0.595 |
| > reference range n (%) | 17 (34.7) | 10 (37.0) | 14 (48.3) | 0.479 |
| Platelets (×10^3^/μL, ref 150-400) | 277.5 (241.8 - 328.8) | 283.0 (256.0 - 330.0) | 298.0 (242.0 - 361.3) | 0.548* |
| WBC (×10^3^/μL, ref 4-10) | 7.7 (6.0 - 9.4) | 7.5 (6.1 - 10.3) | 7.8 (6.5 - 8.9) | 0.974* |
| Neutrophils (×10^3^/μL, ref 1.5-7.5) | 4.4 (3.3 - 6.9) | 5.4 (3.8 - 7.3) | 4.8 (3.8 - 6.6) | 0.594* |
| Lymphocytes (×10^3^/μL, ref 1-3.5) | 2.1 (1.9 - 2.5) | 2.4 (1.7 - 2.8) | 2.2 (1.9 - 2.4) | 0.715* |
| Monocytes (×10^3^/μL, ref 0.1-1) | 0.6 (0.5 - 0.7) | 0.5 (0.4 - 0.6) | 0.6 (0.4 - 0.7) | 0.428 |
| NLR | 2.1 (1.6 - 2.8) | 2.1 (1.8 - 3.0) | 2.2 (1.9 - 2.5) | 0.929 |
| PLR | 124.8 (104.0 - 165.2) | 127.2 (105.7 - 180.9) | 142.4 (113.9 - 166.8) | 0.635 |
| LMR | 3.9 (2.9 - 5.1) | 4.3 (3.7 - 5.7) | 3.8 (3.0 - 4.7) | 0.446 |
| SII | 639.5 (359.4 - 745.2) | 669.2 (535.6 -1038.8) | 692.1 (454.4 - 865.4) | 0.490* |
| ALP (U/L, ref <98) | 78.5 (63.3 - 94.8) | 76.0 (59.8 - 96.3) | 83.0 (67.0 - 95.0) | 0.601* |
| P1NP (µg/L, ref <59) | 47.0 (34.5 - 58.0) | 39.5 (27.3 - 53.0) | 40.0 (26.0 - 59.0) | 0.266* |
| CTx (µg/L, ref <0.573) | 0.214 (0.165 - 0.308) | 0.187 (0.118 - 0.315) | 0.220 (0.154 - 0.315) | 0.373 |

***D:*** *Stratified for disease duration*

Legend: CI, confidence interval; CTx, beta-crosslaps; ESR, erythrocyte sedimentation rate; CRP, C-reactive protein; NLR, neutrophil-lymphocyte ratio; PLR, platelet-lymphocyte ratio; P1NP, procollagen 1 N-terminal propeptide; SII, Systemic immune Inflammation Index.
data reported as median (IQR), * parametric test used for comparative analysis
