# Supplementary material for: Immunological Characterization of Chronic Nonbacterial Osteomyelitis (CNO) in Adults: A Cross‐Sectional Exploratory Study
Source: JBMR Plus. 2023 Sep 11;7(12):e10818. doi: 10.1002/jbm4.10818 (PMC10731106; doi:10.1002/jbm4.10818)
Supplement: Supplementary file 2 — Table S2. Clinical characteristics of cohort subset evaluated for cytokine profiles and RANKL/OPG. [file JBM4-7-e10818-s002.docx]

**Supplementary table 2:** Clinical characteristics of cohort subset evaluated for cytokine profiles and RANKL/OPG

|  | **CNO (n=33)** | **Controls (n=8)** | **Osteoporosis (n=22)** | |  | ***p*** |
| --- | --- | --- | --- | --- | --- | --- |
| Gender, female n (%) | 32 (97.0) | 5 (62.5) | 16 (72.7) |  | | 0.019 |
| Age, mean±SD | 44.8±13.0 | 51.4±12.1 | 62.7±12.3 |  | | 0.000 |
| Active smoker n (%) | 6 (18.2) | 1 (12.5) | 1 (4.5) |  | | 0.793 |
| NSAID use n (%) | 18 (54.5) | 1 (12.5) | 1 (4.5) |  | | 0.032 |
| Disease duration, mean±SD | 7.3±4.0 | - | - |  | | - |
| Clinical subtype n (%) |  |  |  |  | |  |
| CNO | 24 (72.7) |  |  |  | |  |
| CNO + PPP/psoriasis and/or arthritis | 9 (27.2) |  |  |  | |  |

Legend: CNO; chronic nonbacterial osteomyelitis, NSAID; non-steroidal anti-inflammatory drug
