# Supplementary material for: Immunological Characterization of Chronic Nonbacterial Osteomyelitis (CNO) in Adults: A Cross‐Sectional Exploratory Study
Source: JBMR Plus. 2023 Sep 11;7(12):e10818. doi: 10.1002/jbm4.10818 (PMC10731106; doi:10.1002/jbm4.10818)
Supplement: Supplementary file 3 — Table S3. Results of immunoassays for Th1/2 and Th17‐derived cytokines and ELISA for RANKL and OPG in CNO patients, healthy controls and osteoporosis patients. [file JBM4-7-e10818-s003.docx]

**Supplementary table 3:** Results of immunoassays for Th1/2 and Th17-derived cytokines and ELISA for RANKL and OPG in CNO patients, healthy controls and osteoporosis patients

|  | **Detection range (pg/mL)** | **CNO (n=33)** | **Healthy controls (n=8)** | **Osteoporosis (n=21)** | ***p*** |
| --- | --- | --- | --- | --- | --- |
| **Th1/2-derived cytokines (pg/mL)** |  |  |  |  |  |
| IFN-γ | *1.64-1340* | 2.1±1.9 | 1.9±1.4 | 5.0±2.8 | **<0.0001** |
| IL-10 | *0.0615-384* | 0.2±0.1 | 0.2±0.1 | 0.2±0.1 | 0.324 |
| IL-12p70 | *0.212-480* | 0.3±0.2 | 0.4±0.1 | 0.4±0.2 | **0.021** |
| IL-13 | *0.667-503* | 0.7±0.5 | 0.9±0.3 | 0.9±0.5 | 0.138 |
| IL-1β | *0.177-613* | 31/33 < LLOD | All < LLOD | All < LLOD | - |
| IL-2 | *0.0533-1440* | 0.2±0.3 | 0.4±0.3 | 0.3±0.2 | 0.061 |
| IL-4 | *0.0252-266* | All < LLOD | All < LLOD | All < LLOD | - |
| IL-6 | *0.181-739* | 0.9±0.8 | 0.8±0.5 | 1.0±0.7 | 0.753 |
| IL-8 | *0.0855-658* | 7.1±2.7 | 6.0±1.6 | 9.0±4.0 | **0.022** |
| TNF-α | *0.0822-337* | 0.8±0.2 | 0.8±0.3 | 0.9±0.3 | 0.057 |
| **Th17-derived cytokines (pg/mL)** |  |  |  |  |  |
| IL-21 | *2.27-932* | All < LLOD | All < LLOD | All < LLOD |  |
| IL-31 | *0.102-970* | 30/33 < LLOD | All < LLOD | 20/21 < LLOD | - |
| IL-17A | *1.72-3110* | 31/33 < LLOD | All < LLOD | All < LLOD | - |
| IL-22 | *0.156-521* | 0.6±0.6 | 0.5±0.4 | 0.7±0.6 | 0.679 |
| IL-23 | *0.912-5110* | All < LLOD | All < LLOD | All < LLOD | - |
| IL-27 | *24.2-19900* | 754±339 | 768±469 | 681±351 | 0.565 |
| MP3α | *0.39-506* | 5.3±5.0 | 2.9±1.9 | 6.5±4.5 | 0.132 |
| **Bone markers (pg/mL)** |  |  |  |  |  |
| RANKL | *78.1-5000* | 22/33 < LLOD | 5/8 < LLOD | 15/21 < LLOD | - |
| OPG | *62.5-4000* | 891.6±252.9 | 915.0±286.8 | 951.1±354.7 | 0.182 |

Legend: IFN, interferon; IL, interleukin; TNF, tumor necrosis factor; RANKL, receptor activator of nuclear factor kappa beta; OPG, osteoprotegerin; data reported as mean±SD, p-values obtained with parametric/non-parametric tests according to data type.
